# HPV 16 E7 alters translesion synthesis signaling

**DOI:** 10.1186/s12985-022-01899-8

**Published:** 2022-10-20

**Authors:** Sebastian O. Wendel, Avanelle Stoltz, Xuan Xu, Jazmine A. Snow, Nicholas Wallace

**Affiliations:** Division of Biology, Kansas State University, Manhattan, KS 66506 USA

**Keywords:** Human papillomavirus, Cervical cancer, Replication stress, Translesion synthesis

## Abstract

A subset of human papillomaviruses (HPVs) are the cause of virtually every cervical cancer. These so-called “high-risk” HPVs encode two major oncogenes (HPV E6 and E7) that are necessary for transformation. Among "high-risk” HPVs, HPV16 causes most cervical cancers and is often used as a representative model for oncogenic HPVs. The HPV16 E7 oncogene facilitates the HPV16 lifecycle by binding and destabilizing RB, which ensures the virus has access to cellular replication machinery. RB destabilization increases E2F1-responsive gene expression and causes replication stress. While HPV16 E6 mitigates some of the deleterious effects associated with this replication stress by degrading p53, cells undergo separate adaptations to tolerate the stress. Here, we demonstrate that this includes the activation of the translesion synthesis (TLS) pathway, which prevents replication stress from causing replication fork collapse. We show that significantly elevated TLS gene expression is more common in cervical cancers than 15 out of the 16 the other cancer types that we analyzed. In addition to increased TLS protein abundance, HPV16 E7 expressing cells have a reduced ability to induct a critical TLS factor (POLη) in response to replication stress-inducing agents. Finally, we show that increased expression of at least one TLS gene is associated with improved survival for women with cervical cancer.

**Supplementary Information:**

The online version contains supplementary material available at 10.1186/s12985-022-01899-8.

## Background

The manuscript describing the papillomavirus episteme lists 227 different human papillomaviruses (HPVs) in this family of double-stranded circular DNA viruses [1]. A more recent review from the senior author of the first manuscript lists 450 different HPV types, indicating that more HPVs have been identified [2]. This large family of viruses is grouped by sequencing homology into five subgenera and infects mucosal and cutaneous epithelia. While members of each of these genera can cause disease, the severity of the disease differs widely among HPVs. This is particularly true for the alpha genus of HPV, which contains viruses that cause benign warts and deadly cancers [3]. Based on their relative pathogenic potential, alpha genus HPVs are differentiated into “high-risk” and “low-risk” subgroups. Because the work described here focuses exclusively on “high-risk” HPVs, we will refer to them simply as HPVs moving forward. HPVs are responsible for malignancies that kill over 300,000 people each year. However, not all HPVs contribute equally to this grim statistic. HPV 16 and HPV 18 cause more tumors than the other “high-risk” HPVs combined.

HPV-associated tumors occur throughout the anogenital tract and in the oropharyngeal tract [3–6]. Among tumors caused by HPV infections, cervical cancers are unique in that HPV infections cause virtually every cervical cancer. HPV-related carcinogenesis is a decade-long process that requires continuous expression of the E6 and E7 oncogenes [7]. During this process, the cell cycle is dysregulated, DNA-damage repair pathways are highjacked and the cellular genome is destabilized [8]. The viral oncogenes likely contribute directly to the loss of genome fidelity as cells expressing E6 and E7 have a higher frequency of spontaneous double-strand breaks [9]. This is likely due to disruption in DNA repair mechanisms as HPV oncogenes impair the base excision repair [10], nucleotide excision repair [11], Fanconi anemia pathway [12], homologous recombination [13, 14], non-homologous end joining [15] and microhomology-mediated end joining [15] pathways. Likely due to their reduced ability to repair damaged DNA, HPV-related tumors are typically responsive to platinum-based therapies, such as cisplatin [16]. Cisplatin acts by causing DNA lesions that themselves cause replication stress. This provides some specificity for targeting tumor cells that are less likely to pause cell cycle progression to address the DNA lesion. We recently demonstrated in vitro that cervical cancer cells can acquire cisplatin resistance by increasing the expression of polymerases involved in the translesion synthesis or TLS pathway [17–19]. These TLS polymerases let cells tolerate higher concentrations of cisplatin by allowing replication forks to bypass cisplatin-induced DNA lesions and thus avoid cisplatin-induced replication stress.

The HPV lifecycle is dependent on the induction of replication stress responses, including activation of ataxia telangiectasia and Rad3-related (ATR) kinase [20, 21]. This replication stress is primarily induced by expression of the HPV16 E7 [20, 20, 22, 23]. Cells respond to HPV16 E7-induced replication stress by activating several replication stress tolerance mechanisms. The primary means by which HPV16 E7 is known to induce replication stress tolerance is by epigenetic modifications to the host genome [23]. However, the extent that cells respond to HPV16 E7-associated replication stress by increasing TLS activity is unclear. We have shown that TLS gene expression is often elevated in cervical cancers [19]. Given that TLS responds to replication stress and that replication stress is common in tumors, it is not surprising that these genes are highly expressed in cervical cancer. But how does the frequency of increased TLS gene expression in cervical cancer compare to the frequency in other tumor types?

In this manuscript, we analyzed TLS gene expression in cervical cancer data from the cancer genome atlas (TCGA) database [24] and cervical cancer cell lines. To provide mechanistic insight, we examined the impact of HPV16 E7 wild type in primary keratinocyte cell lines. This showed that HPV 16-E7 increased the abundance of TLS proteins and as well as a post-translational modification of PCNA indicative of TLS pathway activation. This appears to be at least partially dependent on RB-degradation as the expression of a mutant HPV16 E7 that cannot bind Rb had a reduced ability to induce TLS. Finally, we used the TCGA database to show that increased TLS gene expression occurs more often in cervical cancers than in most other cancer types and that this is associated with improved patient outcomes.

## Methods

### Heatmap

The hierarchically clustered heatmap was plotted using the *cluster map* function in Python Seaborn module. The row order (cancer) was based on the color bar indicating the increasing rate of TLS genes for each cancer type. The columns (TLS genes) were permuted using the hierarchical clustering method (Weighted Pair Group Method with Arithmetic Mean) to get a structured gene pattern with the dendrogram on the top (https://docs.scipy.org/doc/scipy/reference/generated/scipy.cluster.hierarchy.linkage.html).

### cBioPortal and gene ontology analysis

Web-based software on www.cbioportal.org was used to perform the Kaplan Meyer, gene expression, promoter methylation, and copy number alteration analyses of data in TCGA database. Gene ontology analysis was performed using Gene Ontology enRIchment anaLysis and visuaLizAtion tool (GOrilla) on RNAseq data from the cancer cell line encyclopedia and TCGA databases [25–28]. The 50 cancers examined in Additional file 1: Fig. S1B are Adrenocortical Carcinoma, Hepatobiliary Cancer, Bladder Urothelial Carcinoma, Mucinous Adenocarcinoma of the Colon and Rectum, Rectal Adenocarcinoma, Colon Adenocarcinoma, Breast Cancer, Anaplastic Astrocytoma, Anaplastic Oligoastrocytoma, Oligoastrocytoma, Astrocytoma, Oligodendroglioma, Glioblastoma Multiforme, Pheochromocytoma, Miscellaneous Neuroepithelial Tumor, Cervical Cancer, Esophagogastric Cancer, Tubular Stomach Adenocarcinoma, Stomach Adenocarcinoma, Diffuse Type Stomach Adenocarcinoma, Mucinous Stomach Adenocarcinoma, Signet Ring Cell Carcinoma of the Stomach, Uveal Melanoma, Head and Neck Squamous Cell Carcinoma, Renal Clear Cell Carcinoma, Chromophobe Renal Cell Carcinoma, Papillary Renal Cell Carcinoma, Hepatocellular Carcinoma, Lung Adenocarcinoma, Lung Squamous Cell Carcinoma, Non-Hodgkin Lymphoma, Acute Myeloid Leukemia, Serous Ovarian Cancer, Pancreatic Cancer, Pleural Mesothelioma, Biphasic Type, Pleural Mesothelioma, Epithelioid Type, Prostate Adenocarcinoma, Melanoma, Cutaneous Melanoma, Soft Tissue Sarcoma, Seminoma, Non-Seminomatous Germ Cell Tumor, Embryonal Carcinoma, Thymoma, Follicular Thyroid Cancer, Papillary Thyroid Cancer, Endometrial Carcinoma, Uterine Endometrioid Carcinoma, Uterine Serous Carcinoma/Papillary Serous Carcinoma, and Uterine Carcinosarcoma/Malignant Mixed Mullerian.

### Cell culture

HeLa (ATCC^®^ CCL-2™) and SiHa (ATCC^®^ HTB-35™) cells were grown in Dulbecco modified Eagle medium (DMEM) supplemented with 10% fetal bovine serum (FBS) and penicillin–streptomycin. Primary Keratinocytes (HFKs) were derived in-house from anonymized medical waste. HFKs were grown in Keratinocyte Growth Medium 2 (PromoCell, Heidelberg, Germany, C-20011), supplemented with penicillin–streptomycin, calcium (0.5 M, 60 μl in 500 ml medium), and SupplementMix (PromoCell, Heidelberg, Germany). Virus for retroviral transduction of E7 and E7ΔDLYC into HFKs was produced in 293 T cells grown in DMEM and retroviral transduction was carried out as previously described [29]. All cell lines were grown at 37C and 5% CO_2_.

### Immunoblots

Lysates were generated and immunoblots were run as previously described [30]. The membranes were then probed using the following antibodies: p-ATM (Cell Signaling Technologies, catalog no. 13050S), ATM (Cell Signaling Technologies, catalog no. 92356S), p-ATR (Cell Signaling Technologies, catalog no. 30632S), ATR (Cell Signaling Technologies, catalog no. 2790S), p-CHK1 (Cell Signaling Technologies, catalog no. 2348S), CHK1 (Cell Signaling Technologies, catalog no. 2360S), ub-PCNA (Cell Signaling Technologies, catalog no. 13439S), p-RPA32 (Cell Signaling Technologies, catalog no. 54762S), RPA32 (Cell Signaling Technologies, catalog no. 52445S), TopBP1 (Santa Cruz Biotechnology, catalog no. sc-271043), POLη (Santa Cruz Biotechnology, catalog no., sc-17770), GAPDH (Santa Cruz Biotechnology, catalog no. sc-47724), Nucleolin (Santa Cruz Biotechnology, catalog no. C23-HRP, sc8031) and Rad6 (Abcam, catalog no. ab31917). After incubation with the corresponding horseradish peroxidase (HRP)-conjugated secondary antibody, cells were visualized using SuperSignal West Femto maximum sensitivity substrate (Thermo Scientific).

### Nucleoside supplementation

HeLa and SiHa cells were seeded at 100,000 cells/well on 6-well plates. 24 h after seeding, the DMEM medium was aspirated and replaced by DMEM media supplemented with 10 mg/L nucleoside stock solution (Biological Industries Israel Beit-Haemek Ltd. 01-343-1D). Cells were grown for 72 h before cell lysates were harvested.

### Hydroxy urea, ultraviolet, and cisplatin treatment

HFKs were seeded on 6-well plates at a density of 100,000 cells/well. After 24 h, the keratinocyte growth medium was aspirated and cells were treated with 0.3 mM hydroxyurea (Thermo Fisher Scientific A10831) or 10 μM cisplatin (Sigma Aldrich 479,306-1G) in keratinocyte growth medium. For the UV treatment, cells were exposed to 5 mJ/cm^2^ UV-C (UV Stratalinker 2400, Stratagene) before fresh keratinocyte growth medium was reapplied. After 4 h cell lysates were harvested.

## Results

### Translesion synthesis gene expression is increased in cervical cancers

**Fig. 1:**
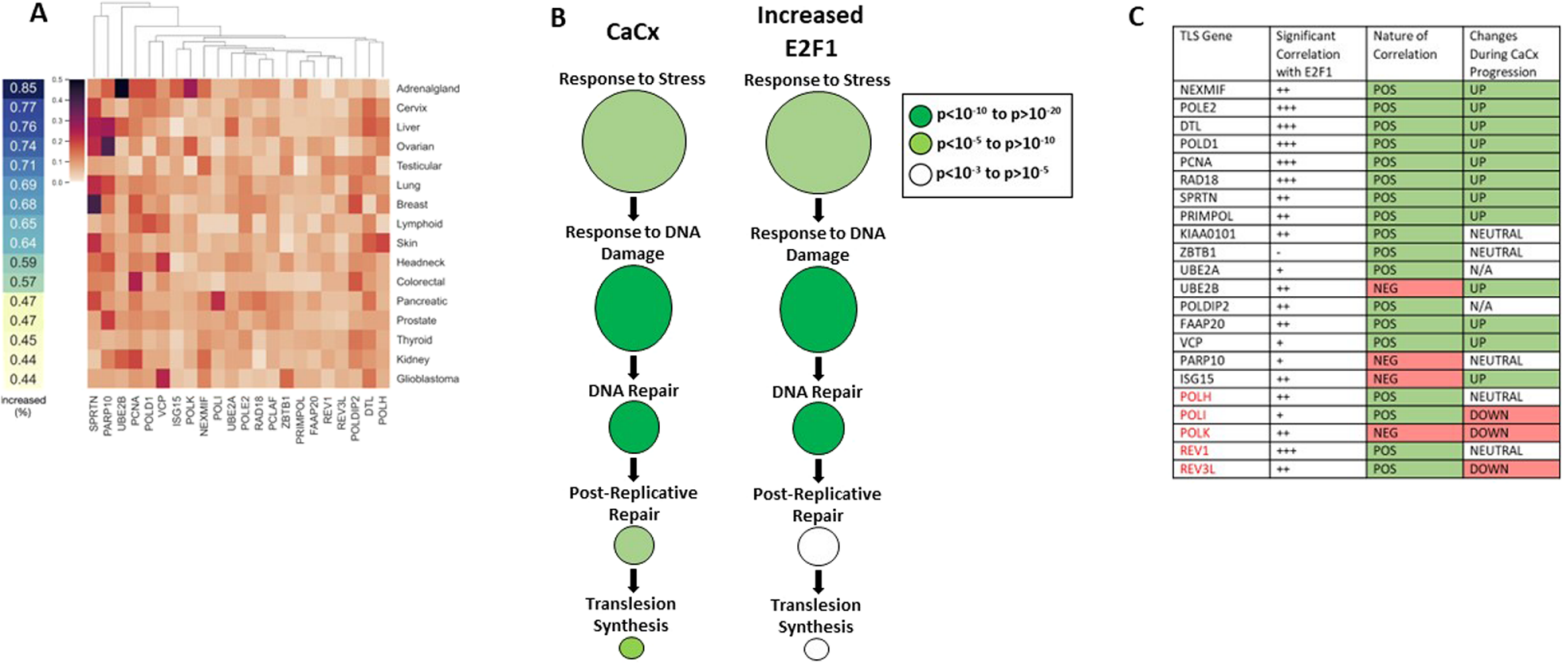
Translesion synthesis gene expression is frequently elevated in cervical cancers compared to other cancer types. **A** Overall frequency with which at least tumors have elevated (z > 2) expression of at least one TLS gene (left). The frequency with which tumors have elevated (z > 2) expression of individual TLS genes (right). Unbiased cluster analysis was used to group individual TLS genes by similarities of gene expression changes. **B** Gene ontology analysis of cervical cancers (CaCx) and cell lines with elevated E2F1 expression (Increased E2F1) showing enrichment of genes involved in the indicated cellular processes. The size of the circle indicates the relative breadth of the cellular process, with large circles indicating broad categories and smaller circles indicating more specific cellular responses. *p*-values are indicated by the color of the circle with darker greens denoting lower p-values than lighter colored circles. **C** This table shows (i) the extent that expression of individual TLS genes correlates with E2F1 expression, (ii) the nature of that correlation, and (iii) how the expression of TLS genes changes with cervical cancer progression. “ − “ indicates that a correlation does not exist or did not reach statistical significance. “ + ”,” +  + ”,” +  +  + ” indicate increasing magnitudes of correlation with E2F1 expression. “POS” indicates the correlation between the expression of the indicated TLS gene and E2F1 is positive. “NEG” indicates the correlation is negative. “Up” indicates that expression of the indicated TLS gene increases as cervical cancers progress from early to later stages of the disease. “Neutral” indicates that these changes are inconsistent (both up and down). “N/A” indicates that this gene was not included in the analyzed data set. “Down” indicates that expression of the indicated gene decreased with disease progression

We have previously reported that the expression of TLS genes is often increased in cervical cancers [19]. To understand the extent that this increased expression occurred more or less frequently than in other cancers, we compared TLS gene expression across 16 tumor types found in the TCGA database [31]. This showed that TLS gene expression more than two standard deviations above the mean (z score > 2) was common in tumors of all types, with an average of 62% of tumors having increased expression of one or more TLS genes (Fig. 1A, Additional file 1: S1A). We also found that increased expression of at least one TLS gene was more common in cervical cancers (77%) than all other tumor types except those occurring in the adrenal gland (85%). Of TLS genes, increased expression of SPRTN, DTL, POLD1, PCNA, and VCP occurred most often in cervical cancers. ZBTB1, REV1, POLI, POLK, and REV3L were least commonly increased. Notably, four of the five genes that are least likely to have elevated expression were TLS polymerases. This is consistent with our prior work demonstrating that HPV16 E6 blocks the induction of POLη in response to replication stress [19]. An analysis of TLS gene expression in 50 different cancer types found a clear correlation between TLS gene expression overall and TLS polymerase expression (Additional file 1: Fig. S1B). In this more expansive analysis, elevated TLS gene expression was similarly more common in cervical cancers (6th out of 50) compared to other cancer types.

Of the two HPV16 oncogenes, HPV16 E7 is more closely linked with replication stress [32]. HPV16 E7 increases replication stress by destabilizing RB-E2F1 complexes and thus dysregulating S-phase entry via E2F1-responsive gene expression [33, 34]. Other RB family members (p130 and p107) are destabilized by HPV E7, which also facilitates S-Phase entry [35], p. 130). To understand the extent that the changes in TLS gene expression could be attributed to increased E2F1 availability, we performed an in silico screen of RNAseq data from 1020 cell lines in the cancer cell line encyclopedia [25]. These cell lines were segregated based on E2F1 expression, allowing us to compare TLS gene expression between the cell lines with and without elevated E2F1 expression. 37 cell lines had elevated E2F1 expression (z-score > 2). This cohort of cell lines was designated “high E2F1 expressing” and compared to the other cell lines to identify differentially expressed genes. We performed gene ontology analysis (via Gene Ontology enRIchment anaLysis and visuaLizAtion tool or GOrilla) on these differentially expressed genes and the CaCx transcriptome after ranking genes by their magnitude of expression (Additional file 1: Fig. S1C). The enriched cellular processes were similar in both cases and included TLS (Fig. 1B). Further analysis found a positive correlation of TLS gene expression with E2F1 expression when the cell lines from the cancer cell line encyclopedia. There was elevated expression of most of these same genes in cervical cancers (Fig. 1C). Because HPV16 E7 expression increases as a function of cervical cancer progression, we determined how the expression of these genes differed in cervical tissues with no evidence of disease, premalignant lesions, and in Stage I-III cervical cancer by interrogating a previously published dataset that we generated by normalizing and combining RNAseq data from multiple publicly available datasets (GSE145976) [19]. The expression of most TLS genes, with the notable exception of TLS polymerases, increased as a function of CaCx progression (Fig. 1C).

### Changes in copy number are a major cause of increased TLS gene expression in cervical cancer

**Fig. 2:**
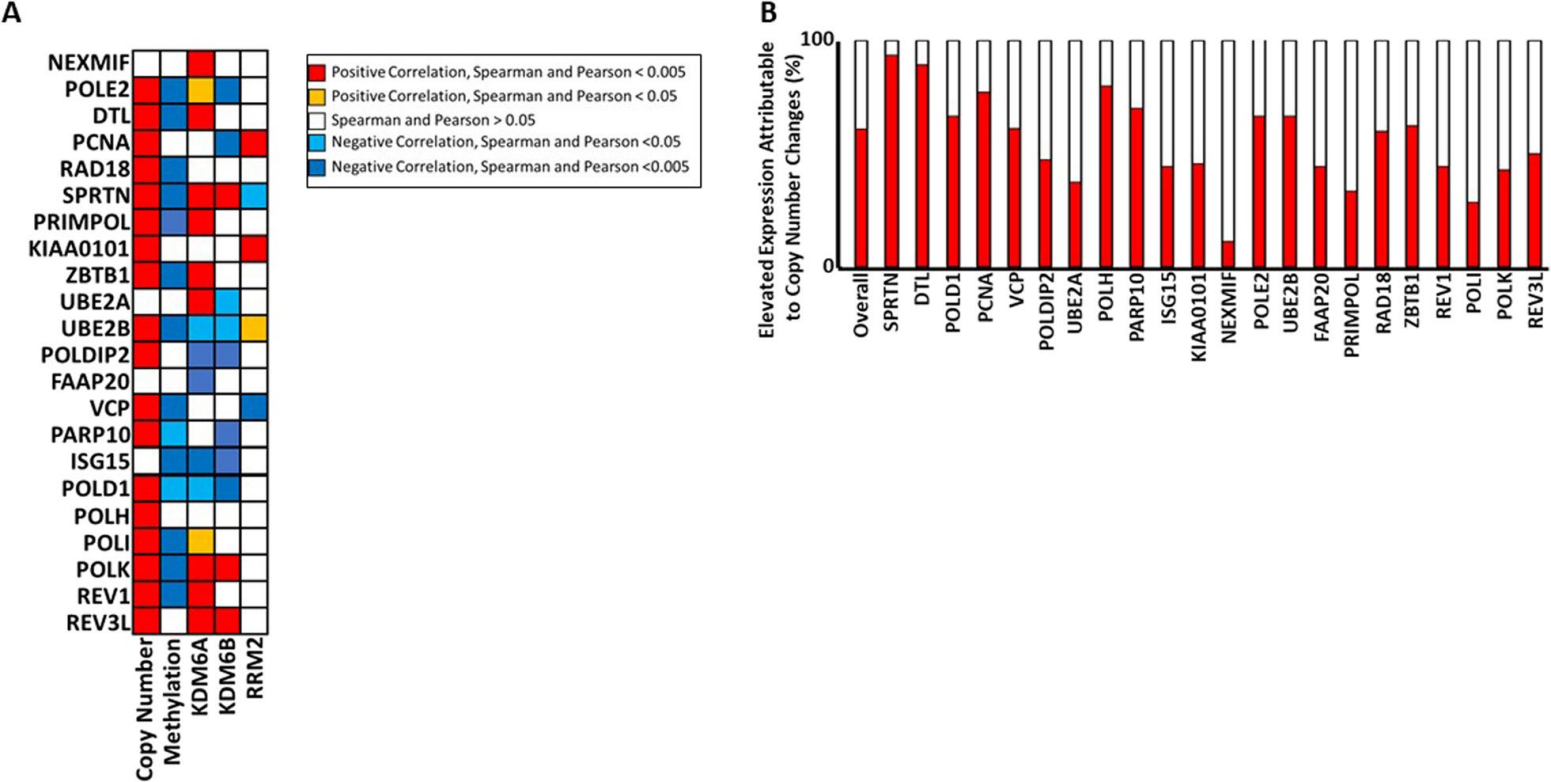
Elevated TLS gene expression is frequently the result of copy number increases. **A** The extent that expression of individual TLS genes significantly correlates with the indicated variable (Copy number, promoter methylations, KDM6A expression, KDM6B expression, and RRM2 expression), and whether this correlation is negative or positive is shown. **B** Bar graphs show the frequency that increases in copy number (red) occur in tumors with elevated expression of the indicated TLS gene

We then defined the extent that the increased expression of TLS genes could be attributed to established determinants of gene expression by analyzing the TCGA database. As expected, copy number alterations showed strong positive with TLS gene expression in cervical cancers. Similarly, promoter methylation negatively correlated with TLS gene expression in these tumors (Fig. 2A). In vitro studies have shown that HPV16 E7 induces two demethylases (KDM6A and KDM6B) resulting in global changes in gene expression [23]. This led us to hypothesize that expression of KDM6A and KDM6B would positively correlate with TLS gene expression. While there were strong correlations between some TLS genes and KDM6A or KDM6B expression, there was an approximately equal frequency of positive and negative correlations (Fig. 2A).

It has also been demonstrated in vitro that HPV16 E7 expression causes increased RRM2 expression. Because RRM2 facilitates de novo synthesis of nucleotides, the increased expression of the protein is believed to help cells tolerate the replication stress caused by HPV16 E7 [20]. As TLS responds to replication stress, we hypothesized that increased RRM2 expression would negatively correlate with TLS gene expression. However, our analysis of the TCGA data did not support this hypothesis (Fig. 2A). RRM2 expression rarely correlated with TLS gene expression in a statistically significant manner. Further, when RRM2 expression significantly correlated with the expression of a TLS gene, it was roughly equally likely to be a positive or a negative correlation. These analyses suggest that KDM6A, KDM6B, and RRM2 help cervical cancer cells tolerate HPV E7-associated replication stress independently of TLS gene expression, highlighting the substantial investment that cells commit to mitigating this stress.

We next determined how often the increased expression of a TLS gene could be attributed to an increased copy number of that gene. This analysis demonstrated that increases in copy number occurred 60% of the times that there was increased expression of the corresponding TLS gene (Fig. 2B). Despite the high overall frequency, there was considerable variation in the influence of copy number alterations on TLS gene expression. Nearly all occurrences of high SPRTN and DTL expression occur in tumors with copy number increases, while the increases in NEXMIF expression rarely occur in tumors that have increased NEXMIF copy number. This suggests that increases in copy number are a major driver of elevated TLS gene expression overall, but that the contribution of copy number increases on TLS gene expression varies among TLS genes. As protomer methylation is a negative correlate of TLS gene expression, we also determined how frequently high promoter methylation (defined as 90–100% of maximal methylation for the gene of interest) occurred in highly expressed TLS genes. As expected, this was rare. High methylation occurred only 0.4% of the times that TLS genes are highly expressed.

### A depleted nucleoside pool contributes to increased TLS gene expression in cervical cancer cells

**Fig. 3:**
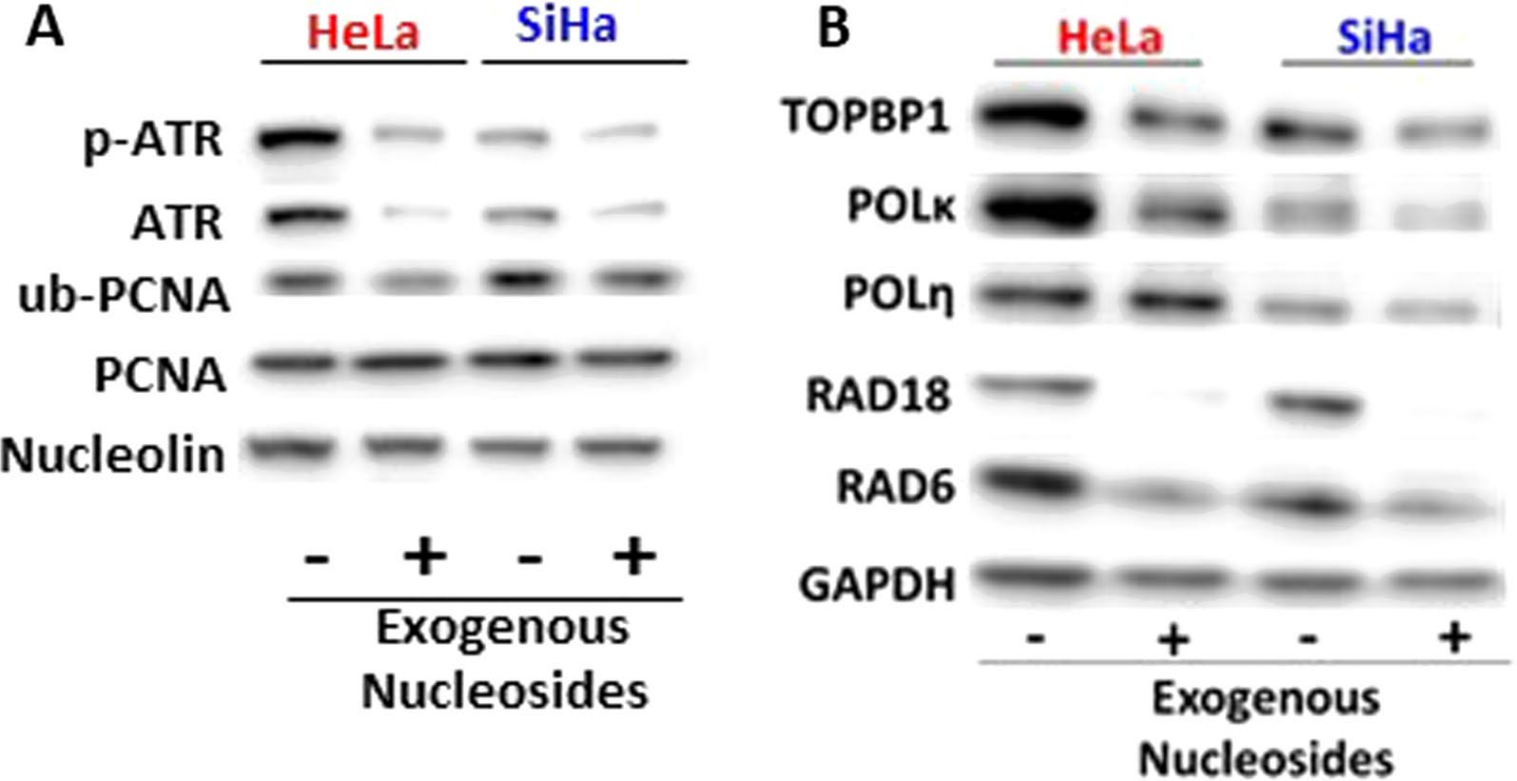
Exogenous nucleoside supplementation decreases replication stress and TLS activation in cervical cancer cell lines. Representative immunoblots of cervical cancer cell lines (HeLa and SiHa) probed for **A** a marker of replication stress (p-ATR) and TLS pathway activation (ub-PCNA) along with total levels of each of these proteins and **B** representative TLS proteins. Whether the cell lines were grown in media supplemented with exogenous nucleosides is indicated below the immunoblot image with “ + ” indicating nucleosides were added and “−“ indicating that they were not. GAPDH and Nucleolin were used as loading controls

We continued to evaluate the expression of TLS genes using two cervical cancer cell lines (HeLa and SiHa). Our published data demonstrate that these cell lines have increased TLS abundance [19]. Combined with our in silico data, this suggests cervical cancer cells utilize the TLS pathway to address replication stress. Thus, we hypothesized that TLS activity could be reduced by lowering replication stress. To test this hypothesis, we grew cervical cancer cells in media with and without additional nucleosides. The addition of nucleosides should remedy replication stress stemming from depleted nucleoside pools and reduce the need for TLS. We harvested whole cell lysates from these cells and conducted immunoblot analysis. ATR is activated via phosphorylation (Thr1989) in response to replication stress [21]. PCNA ubiquitination (Lys 164) is a hallmark of TLS activity [36]. Therefore, antibodies were used to detect total and phosphorylated ATR as well as total and ubiquitinated PCNA or ub-PCNA. We found that the addition of nucleosides to HeLa and SiHa growth media reduced ATR phosphorylation and ub-PCNA abundance (Fig. 3A). Further analysis found that supplemental nucleosides were also able to reduce the abundance of another marker of replication stress (TopBP1) and multiple TLS proteins (POLκ, POLη, RAD18, and RAD6) (Fig. 3B).

### HPV16 E7 causes increases in TLS and replication stress responses

**Fig. 4:**
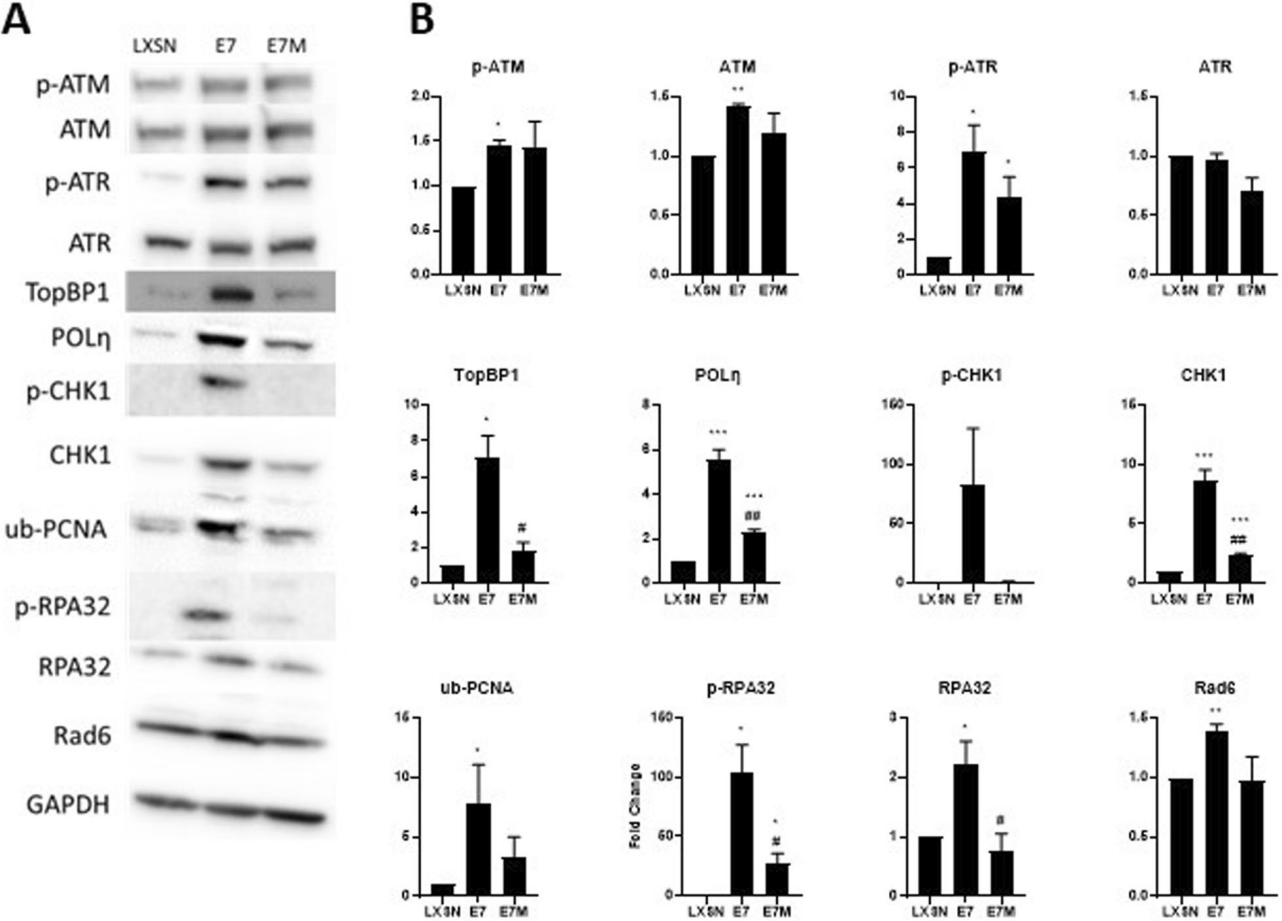
HPV16 E7 increases TLS pathway activation by binding RB. Representative immunoblot of vector control (LXSN) and HPV16 E7 wild type and mutant E7 expressing HFKs probed for **A** replication stress-responsive proteins and TLS proteins and **B** densitometry of these proteins calculated from three individual immunoblots. The bars represent the mean of densitometry from at least three independent repeats. As a result, they may not perfectly match the representative blot shown in A. Error bars denote standard errors of the mean. # denote a statistical difference between HPV16 E7 wild type and HPV16 E7 mutant (#− *p* < 0.05, ##− 0.01). *denotes that there is a statistical difference between LXSN HFKs and the indicated cell line (*− *p* < 0.05, **− 0.01, ***− 0.001). GAPDH was used as a loading control

Together these data suggest that HPV16 E7-associated replication stress leads to increased TLS protein abundance. To test this, we used primary neonatal human foreskin keratinocytes (HFKs) because HPV infections naturally occur in keratinocytes. A lentiviral system allowed us to express HPV16 E7 at physiological levels and compare changes induced by HPV16 E7 to vector only (LXSN) HFKs. This system is widely used to study HPV oncogene biology as it drives HPV oncogene expression at levels accepted to be below those seen in cell lines derived from cervical cancers, where super enhancer elements drive increased oncogene expression [37]. We confirmed the expression of HPV16 E7 in these cells using RB levels as a surrogate marker of HPV16 E7 (Supplemental Fig. 2). Immunoblot analysis demonstrated that HPV16 E7 HFKs had the expected reduction in RB protein levels. Consistent with reports from other groups that high-risk HPV oncogenes increase replication stress markers [13, 20, 38–40], p. 5, [22, 41], we found that HPV16 E7 increased the abundance of replication stress (TopBP1, p-RPA32, p-ATR, p-CHK1) and DSB repair (p-ATM) proteins (Fig. 4). HPV16 E7 also increased TLS activity, as indicated by an increase in ub-PCNA abundance. Further, HPV16 E7 increased the amount of POLη protein that we detected (Fig. 4). The ability of HPV16 E7 to induce replication stress has been linked with its ability to bind and destabilize RB-E2F1 complexes. This binding can be abolished by deleting the residues of E7 that facilitate the interaction with RB (HPV16 E7Δ21-24^DLYC^) [42]. We will refer to this mutant as HPV16 E7ΔDLYC. To determine the extent that HPV16 E7 increased TLS activity and protein abundance by binding RB, we expressed this mutant in HFKs (HPV16 E7ΔDLYC HFKs). Consistent with the mutation abolishing RB destabilization, HPV16 E7ΔDLYC HFKs had approximately the same amount of Rb as LXSN HFKs (Additional file 1: Fig. S2). HPV16 E7ΔDLYC HFKs have generally increased the abundance/activation of TLS proteins (POLη, ub-PCNA, but not Rad6) and replication stress response markers (p-ATR, p-ATM, TopBP1, p-RPA32, but not p-CHK1) compared to LXSN HFKs (Fig. 4). However, the increase was typically less than the increase seen in HPV16 E7 HFKs.

### HPV16 E7 changes cellular responses to replication stress-inducing agents

**Fig. 5:**
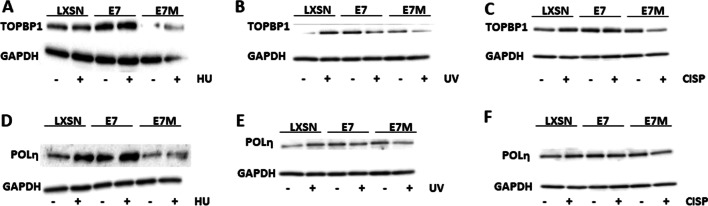
HPV16 E7 alters POLη and TopBP1 induction in response to replication stress-inducing agents. Representative immunoblots of vector control (LXSN) and HPV16 E7 wild type and mutant E7 expressing HFKs probed for TopBP1 (**A–C**) or POLη (**D–F**). Whether these cells were exposed to the indicated replication stress-inducing agent (Hydroxyurea or HU, UV, and Cisplatin or CISP) is indicated by “−“ (not exposed) and “ + ” (exposed). GAPDH was used as a loading control

We next used these cell lines to determine if HPV16 E7 altered cellular responses to replication stress-inducing treatments. Three stressing agents were used, two that caused replication stress by inducing DNA lesions (Cisplatin and UV) and one that depletes nucleoside pools (hydroxyurea or HU). For this analysis, we focused on a single marker of replication stress (TopBP1) and a single marker of TLS (POLη). TopBP1 was chosen because the ability of HPV16 E7 to increase TopBP1 abundance is well established [40, 43, 44]. POLη was chosen because we have previously shown that HPV16 E7 prevented the accumulation of POLη in response to UV damage. In LXSN HFKs, TopBP1 protein levels were increased in response to UV and Cisplatin, but not in response to HU. POLη levels were increased in response to each of the three replication stressing agents in LXSN HFKs (Fig. 5). In contrast, HPV16 E7 prevented the induction of POLη and TopBP1 in response to these stimuli. HPV16 E7ΔDLYC also prevented these increases (Fig. 5).

### Increased TLS gene expression is associated with better survival in people with cervical cancer

**Fig. 6:**
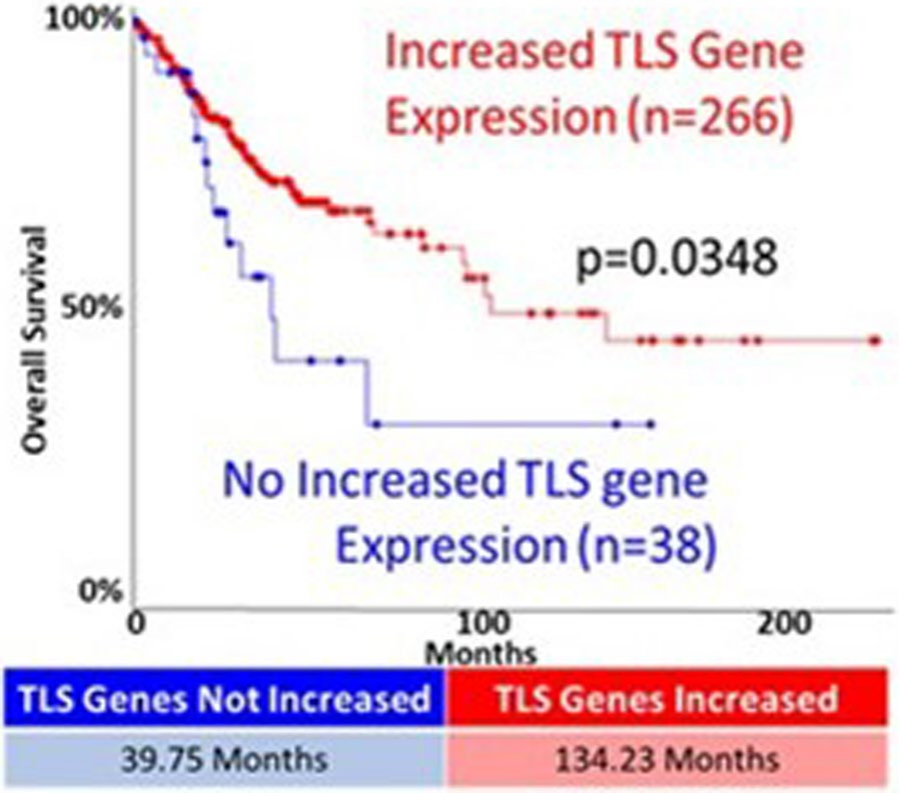
Increased expression of at least one TLS gene is associated with longer cervical cancer survival. Kaplan Meyer analysis of cervical cancers with increased expression of at least one TLS gene (red) compared to cervical cancers without expression of a TLS gene (blue). The median survival for each group is shown below the graph. P-value was calculated by Log Rank Test

We have previously demonstrated that increased expression of TLS polymerases was associated with worse outcomes for people with cervical cancer [19]. To determine if the same was true for TLS genes other than the TLS polymerases (POLH, POLI, POLK, REV1, and REV3L), we segregated the cervical cancers in the TCGA database based on whether they did or did not have increased expression of at least one TLS gene. The rationale for leaving these genes out of the analysis is that we have already shown that when grouped they are associated with worse patient outcomes [19]. As expected most cervical cancers had increased expression of at least one TLS gene. However, this was not associated with poor prognoses. Instead, the patient population with increased TLS gene expression had a median survival of 134.23 months (Fig. 6). In contrast, survival statistics were significantly worse for the group of people without this increased expression (median survival of 39.75 months).

## Discussion

We have previously shown that TLS gene expression is increased in cervical cancers [19]. Here, we continue our examination of translesion synthesis and determine that HPV16 E7, via RB degradation, is a driving force behind increased TLS gene expression. We demonstrated that elevated expression of TLS genes occurs more frequently in cervical cancers than in most other tumor types. Indeed, the only tumor type that we found with more frequently elevated TLS gene expression were tumors occurring in the adrenal gland. We also demonstrated that increases in copy number are a principal driver of increased TLS gene expression and that this is especially true for the TLS genes that are most commonly overexpressed. We used in vitro models of cervical cancer to link these findings to replication stress responses (Fig. 3A, p-ATR, and ub-PCNA) and expression of HPV16 E7 (Fig. 4). Finally, we highlight the importance of examining the TLS pathway in the context of cervical cancer by showing that increased expression of one or more TLS genes is associated with significantly improved patient outcomes (Fig. 6).

We would like to note a few implications of these data: First, the observation that increased copy number was such a common driver of elevated TLS gene expression implies that tumor cells experience a selective pressure to increase TLS gene expression during HPV-mediated transformation. This indicates that HPV16 E7 does not directly drive the increase in TLS gene expression, but rather that HPV16 E7-induced replication stress leads to a selection of cells with a greater ability to tolerate replication stress. The degradation of Rb by HPV16 E7 is a major driver of TLS gene expression. Evidence for this is that HPV16 E7ΔDLYC HFKs have lower TLS protein levels than HPV16 E7 HFKs (Fig. 4B). However, the mutant retains the ability to induce some replication stress responses (note p-ATM in Fig. 4B). Both HPV16 E7 and HPV16 E7ΔDLYC also alter the response to exogenous replication stress. Together, these data suggest that either the mutant does not completely abolish RB destabilization or that HPV16 E7 increases replication stress responses through an RB-independent mechanism(s). Given the extent that HPV16 E7 ΔDLYC has been characterized, we favor the latter interpretation.

We found the way that HPV16 E7 attenuated the cellular response to exogenous replication stress to be interesting. TopBP1 protein abundance increased when cells were exposed to exogenous stressors that physically damage DNA (UV and cisplatin) but did not appreciably rise when grown in media containing HU. HU causes replication stress by depleting nucleoside pools. This suggests that the induction of TopBP1 in response to replication stress differs based on the way the replication stress is induced. In contrast, POLη abundance rose in response to each source of replication stress in LXSN HFKs. The increase in POLη was not seen when either HPV16 E7 and HPV16 E7ΔDLYC was expressed in these cells. As POLη is an error-prone, tightly regulated polymerase, we speculate that there is a maximal amount of POLη that a cell can be expressed. Perhaps, HPV16 E7 expression alone is enough to approach this threshold. Thus, further stimuli are unable to induce further increases. Because this is speculation without evidence that there is a maximal amount of POLη tolerated by cells, we acknowledge that there may be another explanation for these observations.

Our in silico analysis showed that increased expression of TLS genes (excluding TLS polymerases genes) correlated with improved survival in people with cervical cancer (Fig. 6). The in vitro data described here offer a possible explanation. Namely, we show that HPV E7-associated replication stress increases the abundance of TLS proteins (Fig. 4). This is likely clinically relevant as the abundance of TLS proteins in cervical cancer cells can be reduced by reducing replication stress (Fig. 3). We interpret these data as evidence that the expression of TLS genes in cervical cancer likely serves as an indirect metric of the amount of replication stress experienced by that cancer. Cervical cancers are often treated with platinum-based drugs that kill tumor cells by inducing replication stress. Based on these data, we speculate that cervical cancers are easier to treat when they have a high basal level of replication stress, leading to better patient outcomes.

While the increased expression of TLS genes likely indicates that cells are attempting to tolerate replications stress, the scenario is more complicated in the context of cervical cancer. In cervical cancer cells, HPV16 E7 would be expressed in combination with HPV16 E6. HPV16 E6 prevents the TLS pathway from allowing tolerance of replication stress by blocking the induction of the required TLS polymerases [19]. Thus, in cervical cancers, the elevated expression of TLS genes other than TLS polymerases likely indicates a doomed attempt to respond to replication stress by activating the TLS pathway.

There are broader unanswered questions related to our observations as well. For example, the induction of replication stress and DNA repair responses have been linked to the amplification phase of the HPV life cycle. The TLS pathway responds to replication stress and helps mitigate DNA damage, but it is unclear if elevated TLS pathway activity is important for the viral life cycle. Further, we have shown (here and elsewhere) that TLS gene expression in general and TLS polymerase expression specifically are prognostic factors in cervical cancer survival [19]. This suggests that the TLS pathway represents a therapeutic target in cervical cancers.

## Conclusions

High risk α-HPV’s potential to induce genomic instability and specifically its ability to induce spontaneous DNA double strand breaks (DSB) has been extensively described. This presents a double-edged sword for the virus. On the one hand, genomic instability leads to increased expression of host cellular DNA repair factors that HPV requires for its own replication, on the other hand it puts the virus at risk for integration into the host genome which is a dead end. Activation of the TLS pathway by E7 could be part of an effort to maintain the balance between this risk and reward. We further show that E7 expressing cells are unable to respond to additional replication stress by further increasing TLS activation. This could provide a partial explanation for the sensitivity of cervical cancer cells to replication stress inducing chemotherapeutics as well as insight into how a portion of the spontaneously arising DNA double strand breaks detected in HPV oncogene expressing cells occur.

## Supplementary Information

**Additional file 1: Fig.S1.** Expression of TLS genes in cervical cancers. **A** Heat map of each TLS gene in cervical cancer in the TCGA database. The scale bar indicates gene expression values with blue representing a z-score of − 3 and red indicating a z-score of 3. **B** Dot plot of 50 cancer types ranked on the y-axis by the frequency of elevated TLS polymerase expression (z-score >2) and on the x-axis by the frequency of elevated expression of all other TLS genes (z-score >2). Linear regression is shown along with a 95% confidence interval for that regression. The R2 of the line is 0.6562. Cervical cancer is below the line indicating that elevated TLS polymerase expression occurs less often than expected C. Bar graph shows the frequency that each TLS gene had elevated expression (z-score > 2) in cervical cancers. TLS Polymerases are indicated with red bars. All other TLS genes are indicated by black bars. **Fig.S2.** RB abundance in LXSN, E7 wildtype, and E7 mutant HFK cell lines. Representative immunoblot. GAPDH was used as a loading control

## Data Availability

All data and materials are available upon request unless purchased from a commercial entity.
