# Supplementary material for: HPV 16 E7 alters translesion synthesis signaling
Source: Virol J. 2022 Oct 20;19:165. doi: 10.1186/s12985-022-01899-8 (PMC9583550; doi:10.1186/s12985-022-01899-8)

## Slide 1
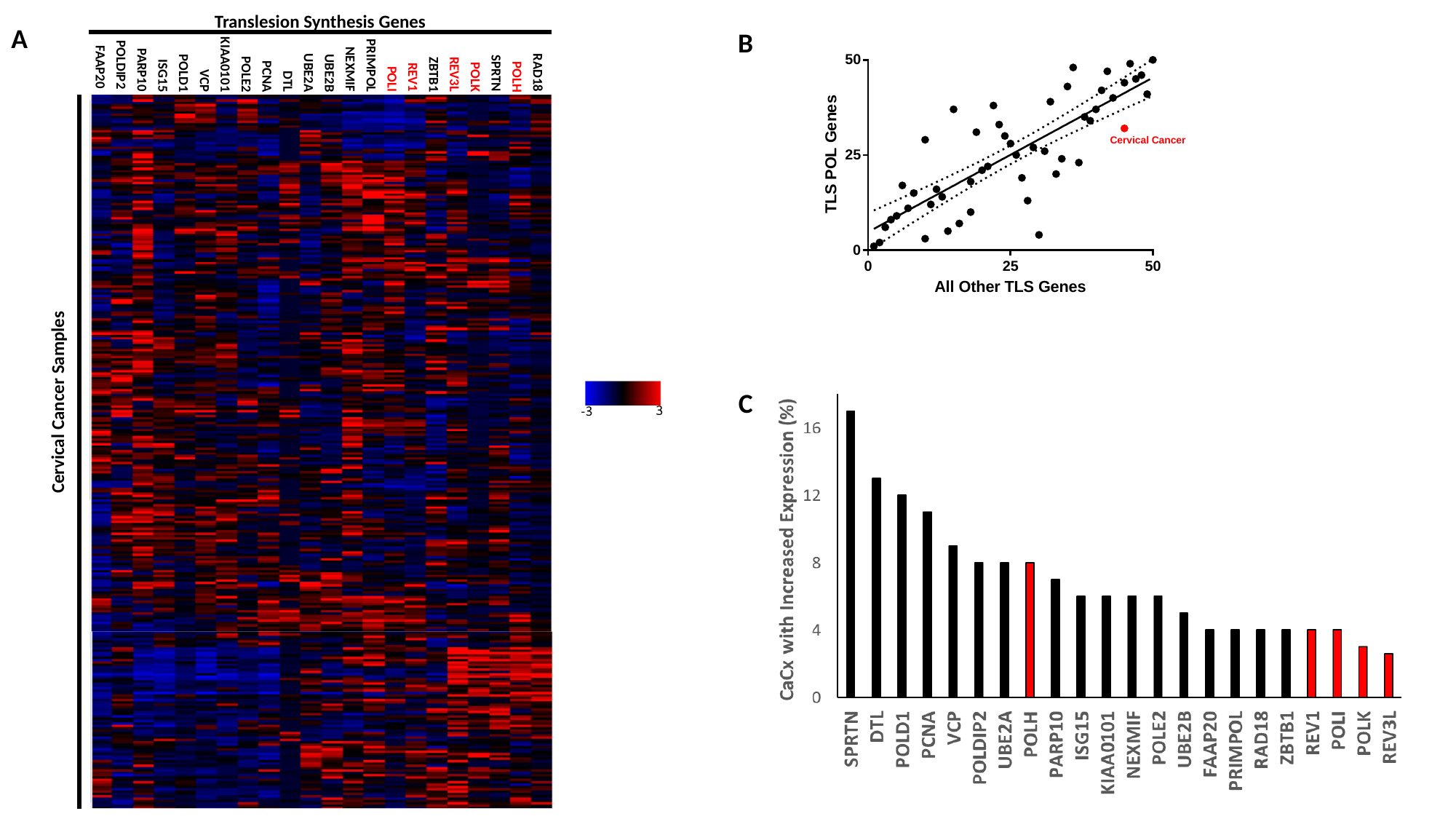

Translesion Synthesis Genes
KIAA0101
PRIMPOL
POLDIP2
FAAP20
NEXMIF
PARP10
UBE2A
RAD18
POLD1
UBE2B
SPRTN
POLE2
ZBTB1
REV3L
ISG15
PCNA
POLH
POLK
REV1
POLI
VCP
DTL
Cervical Cancer Samples
A
B
C

## Slide 2
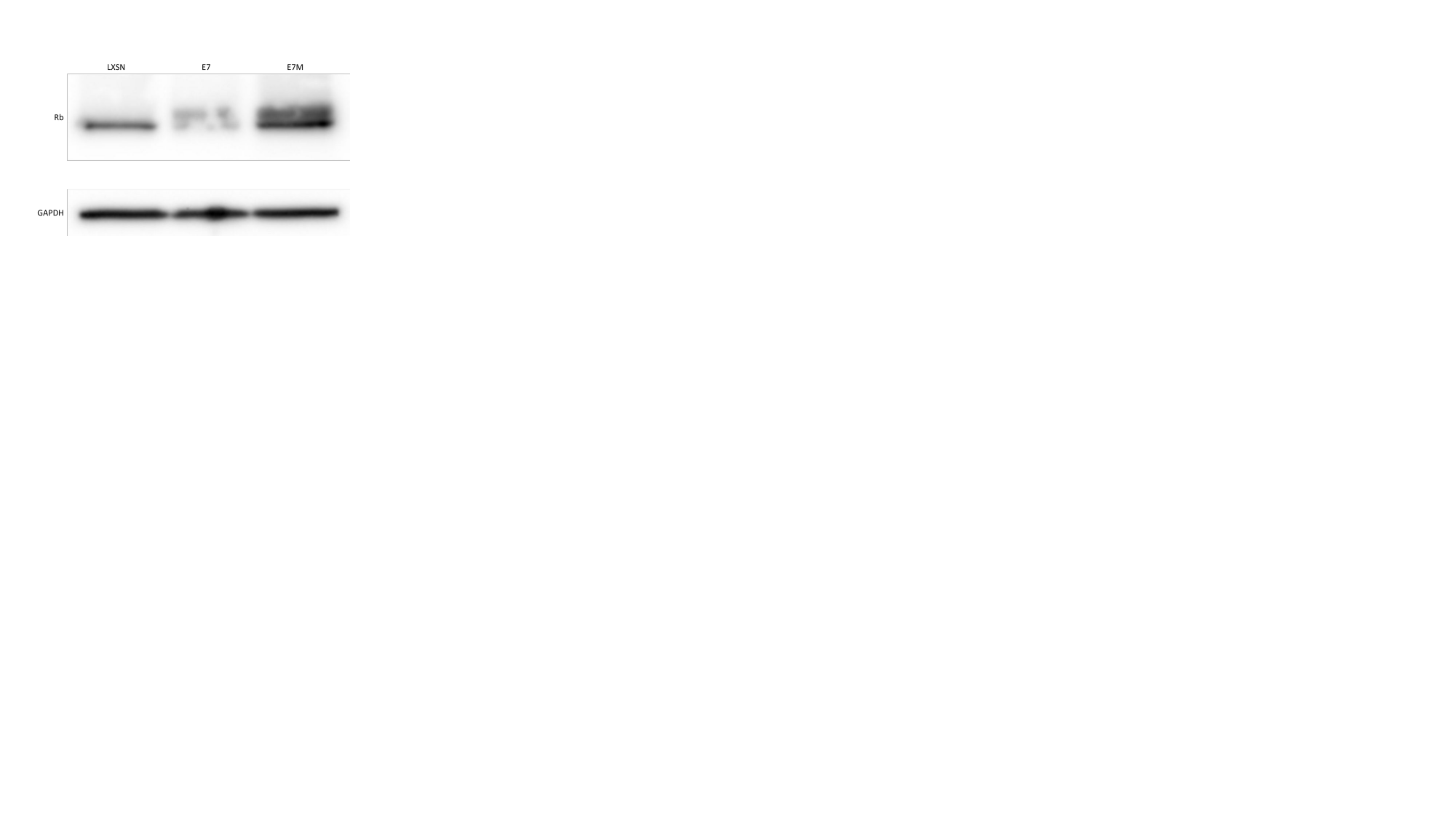

Supplement: Supplementary file 1 — Additional file 1: Fig.S1. Expression of TLS genes in cervical cancers. A Heat map of each TLS gene in cervical cancer in the TCGA database. The scale bar indicates gene expression values with blue representing a z-score of − 3 and red indicating a z-score of 3. B Dot plot of 50 cancer types ranked on the y-axis by the frequency of elevated TLS polymerase expression (z-score >2) and on the x-axis by the frequency of elevated expression of all other TLS genes (z-score >2). Linear regression is shown along with a 95% confidence interval for that regression. The R2 of the line is 0.6562. Cervical cancer is below the line indicating that elevated TLS polymerase expression occurs less often than expected C. Bar graph shows the frequency that each TLS gene had elevated expression (z-score > 2) in cervical cancers. TLS Polymerases are indicated with red bars. All other TLS genes are indicated by black bars. Fig.S2. RB abundance in LXSN, E7 wildtype, and E7 mutant HFK cell lines. Representative immunoblot. GAPDH was used as a loading control [file 12985_2022_1899_MOESM1_ESM.pptx]
